# Sensitive surface plasmon resonance label-free biosensor on a fiber end-facet

**DOI:** 10.1038/s41377-022-01025-x

**Published:** 2022-11-11

**Authors:** Xudong Fan

**Affiliations:** Biomedical Engineering Department, University of Michigan, 1101 Beal Ave., Ann Arbor, MI 48109 USA

**Keywords:** Optics and photonics, Biophotonics

## Abstract

An advanced and cost-effective method was developed to fabricate a high-quality quasi-3D plasmonic crystal biosensor on an optical fiber end-facet.

Label-free optical biosensors have a broad range of applications in biological sciences, drug discovery, and diseases diagnosis^1^. In the past decades, various label-free optical biosensor platforms have been explored and commercialized^1^, such as surface plasmon resonance (SPR) biosensors^2^ (for example, Biacore SPR System from Cytiva), ring resonator biosensors^3^ (for example, Maverick from Genalyte), photonic crystals^4,5^ (for example, Epic BT System from Corning), and bio-layer interferometry (BLI)^6,7^ (for example, Octet BLI Label-Free Detection System from Sartorius), etc., among which SPR is the most sensitive whose sensitivity can be as high as a few nm wavelength shift per nanometer of the biomolecular layer deposited on the SPR sensor surface. However, traditional SPR sensors rely on bulky and complicated optical coupling mechanisms that hinder their applications (such as point-of-care detection). On the other hand, a biosensor fabricated on an optical fiber end-facet has the advantages of easy dip-and-read mechanisms and can significantly simplify the associated fluidic design and liquid handling, as evidenced by the quick and broad adoption of Octet BLI systems. However, BLI-based biosensors usually suffer from very low Q-factors, which limits their biomolecular detection limit. Therefore, the integration of a highly sensitive SPR sensor with a reasonably high Q-factor onto a fiber end-facet becomes very attractive. A long-standing challenge to these devices has been their high fabrication costs and very low signal-to-noise ratios compared to the traditional SPR configurations due to the low coupling efficiencies between the fiber end-facet SPR structures and the fiber-guided light.

In an article published in this issue^8^, Tian Yang’s team and collaborators, based upon their unique work on surface plasmon polariton (SPP) crystal microcavities on single-mode fiber end-facets first reported in 2016^9^, now invented a quasi-3D structure consisting of an SPP-crystal microcavity and a Fabry-Pérot etalon (see Fig. 1). The Fano resonance of the 3D structure shows a Q-factor ~100 and a coupling efficiency of ~50%, resulting in a refractive index detection limit on the order of 10^−7^ RIU (refractive index units), and a potential to detect biomolecules down to the ng/mL range in bulk solution. In addition, the team invented an SPP-tunneling interface that allows the 3D structures to be stripped off from glass substrates with low adhesion, so that the devices can be efficiently produced with high quality by transferring and aligning the SPR structures onto the fiber end-facets. These fiber-tip SPR devices reach the milestone of an SNR comparable to the commercial prism-coupled ones while having the advantages of ease of operation.

**Fig. 1:**
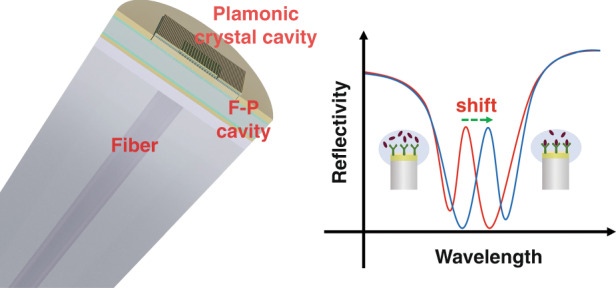
A quasi-3D plasmonic-dielectric hybrid cavity on a single-mode optical fifiber’s end-facet (left) and using its Fano resonance for high signal-to-noise ratio biosensing (right)

The immediate follow-up work would be to test sensor’s detection limit for the biomolecules captured to its surface and compare its performance with a traditional SPR sensor and a BLI sensor. In addition, how to mass produce the sensors with a high yield and high reproducibility should also be addressed in the future to translate this technology to industries and make it commercially competitive.

Overall, the current work sheds light on future fiber-tip plasmonic devices for which we can imagine that a plethora of 3D nanostructures are mounted on fiber end-facets to improve performance and achieve new functions with unprecedented top-down fabrication capabilities.
